# Does household help prevent loneliness among the elderly? An evaluation of a policy reform in the Netherlands

**DOI:** 10.1186/s12889-018-6004-6

**Published:** 2018-09-10

**Authors:** Jelena Arsenijevic, Wim Groot

**Affiliations:** 1 Department of Health Services Research; CAPHRI, Maastricht University Medical Center; Faculty of Health, PO Box 616, 6200 MD Maastricht, The Netherlands; 2 Medicine and Life Sciences, Maastricht University, PO Box 616, 6200 MD Maastricht, The Netherlands; 3 Top Institute Evidence-Based Education Research (TIER), Maastricht University, Maastricht, The Netherlands; 4 Utrecht University School of Governance, Utrecht, The Netherlands

## Abstract

**Background:**

Household help is a community intervention in the Netherlands. Its primary goal is to provide professional help in doing domestic work. A secondary goal of the intervention is to alleviate loneliness. In 2007, a major health care reform and budget cut changed household help. After the reform alleviating loneliness is no longer an aim of the intervention. In this study we evaluate the effects of the policy change in household help on loneliness among older adults in the Netherlands.

**Methods:**

We use SHARE data collected during the period 2004–2013 to compare levels of loneliness among older adults in the Netherlands and those in 9 other European countries (Austria, Belgium, Germany, Denmark, Italy, France, Sweden, Spain and Switzerland). We use a synthetic control method (SCM) based on aggregate data. To check the robustness of our results we also apply a difference-in-differences (DiD) estimator that allows us to estimate the effects of policy changes using individual level data.

**Results:**

In 2004, the prevalence of loneliness ranged between 6.5% in the Netherlands and 15.4% in Italy. Loneliness increased with time for all observed countries. The increase between 2004 and 2013 was highest in France − 30.2%, Italy – 33. 4% and Belgium – 25.0%. The level of loneliness among older adults in the Netherlands increased after 2011. However, it is still lower than in other countries. There was no statistical significant difference in age between waves (67.36 ± 13.7 before 2011 to 68.55 ± 9.24 after 2011, *p* = 0.01). Based on the DiD estimator, there is no statistically significant difference in the incidence of loneliness between older adults in the Netherlands and those in the control countries.

**Conclusion:**

Our results do not suggest that the policy change and budget cut in 2007 on household help has had an effect on loneliness. In absolute numbers, the prevalence of loneliness has increased since 2011, however we find no evidence that this can be attributed to the policy change.

**Electronic supplementary material:**

The online version of this article (10.1186/s12889-018-6004-6) contains supplementary material, which is available to authorized users.

## Background

Loneliness is a subjective feeling where one perceives a negative discrepancy between actual and desired contacts both in quantity and quality [1, 2]. Evidence shows that loneliness increases with age and can have significant negative effects on physical and mental health among elderly [5, 6]. Over the past years the prevalence of loneliness among older adults appears to have increased, particularly among people older than 75 and those with movement difficulties. To reduce loneliness, many European countries have developed health promotion interventions [8]. Those interventions can be divided into individual (one-to-one) based programs (befriending –individual house visits once per month) and community based interventions - where community resources are used to support older adults and their needs [9, 10]. Loneliness among older adults is usually related to life-changing events such as the loss of partner, retirement or reduced mobility due to ageing or illness. Older individuals are less able to go out to meet people and to take actions to prevent loneliness. They are more likely to be bound to their house and dependent on who comes to see them rather than to go out and meet people. This may make community based interventions a better choice in preventing loneliness among older adults. Community based interventions are usually based on nation-wide policies and combine approaches to tackle loneliness with other forms of help and support for older adults [11]. One community intervention in the Netherlands that as a primary goal has providing help to older adults, but also aims to decrease loneliness is household help (‘huishoudelijke hulp’). Household help was first introduced at the end of the 1960’s under the Dutch law known as the AWBZ (General Law on Exceptional Medical Expenses). In 2007, a new law known as the Wmo (Wet maatschappelijke ondersteuning) was introduced. The policy on household help was transferred from AWBZ to Wmo and it was changed. We present the main characteristics of household help policy before and after 2007 in Table 1.

**Table 1:** Description of household help (intervention) before and after 2007

	Household help before 2007	Household help after 2007
Regulation	Regulated by AWBZ (General Law on Exceptional Medical Expenses)	Regulated by Wmo (Wet maatschappelijke ondersteuning)
Goal of AWBZ	To provide necessary domestic help and enable older people to continue living independently in their own house	The main goal of the new law was to provide help and support to ensure that everyone could participate in society [12]
Who is responsible for service allocation	Household help was obtained based upon a care allocation by the Centraal Indicatie Orgaan Zorg (CIZ). CIZ were under regional health centers	It is also regulated by CIZ, but CIZ are now under regulation of local municipalities
Services included	Cleaning, cooking, washing but also personal care and support for emotional needs [12].	Mostly cleaning
Eligibility and entitlements	*Right to care:* everyone who met the eligibility criteria was entitled to an amount of care The entitlement to a number of hours of care was not based on normative requirements but rather reflected the client’s need for help [12]. The services were mostly used by older adults but also by younger people with chronic physical or physiological diseases.	Right to compensation: The right to compensation gave the municipalities the freedom to develop their own policy regarding service provision [13] This means that municipalities can develop tailor-made solutions for each individual depending on their individual circumstances. With Wmo household help is provided only to persons who have no other ways to organize help or support

Household help in the AWBZ was seen by policy makers as an effective tool not only to help but also to decrease loneliness [12]. Reforms in 2007 that have been extended in further reforms and the total abolishment of the AWBZ in 2015, were controversial before their introduction. The introduction of the Wmo was accompanied by a budget cut of appr. €300 million on a total budget for household help of appr. € 1.500 million. However, client satisfaction surveys conducted after the introduction of the Wmo in 2007 showed that clients were generally satisfied with the care they received. As a result of the reform and the budget cuts many professional household helpers lost their job. Those who kept their jobs saw their work become more focused on house cleaning with less time available for social contacts with the clients and monitoring of the social and mental well-being of clients.

There have been some attempts to evaluate the effects of the reform and the transition from household help provided under AWBZ regulations to the Wmo [13]. However, this evaluation had some methodological limitations. For example, one of the studies used only descriptive measures to compare the effects [13] and did not take in account that participants who used household help before 2007 might differ from those who used the services after 2007 in many (un)observed characteristics. Those characteristics can influence the estimated effects of the changes in household help. This situation is known as a selection bias problem [14]. Furthermore, previous studies did not evaluate the effects that the policy changes have had on loneliness among older adults.

The aim of this study is to evaluate the effects of the policy change in household help on loneliness among older adults in the Netherlands in 2007. To overcome selection bias problems, we use two different methods. First, we apply a synthetic control method (SCM) that allows us to control for selection bias but also to perform a country comparison [15]. Some authors have argued that there are cross country differences in loneliness among older adults and that they can be attributed to differences in cultural expectations [3]. People from Southern European countries are expected to have more social contacts than people from the North [4, 16, 17]. The SCM allows us to create a synthetic control group from different countries that are most similar to the treatment group in the Netherlands before the policy change [15]. In this way, we overcome the cultural bias that might influence the level of loneliness among older adults in different countries. The main disadvantage of this method is the use of aggregated data and that this might lead to less statistical power [18]. In order to check the robustness of our results we also apply a difference-in-differences (DiD) estimator that allows us to estimate the effects of policy changes using individual level data. For this we use SHARE (The Survey of Health, Ageing and Retirement in Europe) data collected during the period 2004–2013 [15, 19].

## Methods

### Data

SHARE data are longitudinal data collected at five time points 2004 (wave 1), 2006 (wave2), 2008 (wave 3), 2011 (wave 4) and 2013 (wave 5). We exclude wave 3 and wave 2. Wave 3 is focused on participants’ lifestyle and does not provide information relevant for this study. Wave 2 provides information about loneliness using one-single item with two level answers: yes and no, while the other waves use a different measurement with multi-level answers. Data for all included waves are available for 10 different European countries, namely: Austria, Germany, Sweden, the Netherlands, Spain, Italy, France, Denmark, Switzerland and Belgium. Since the aim of this study is to explore the effects of a policy change on loneliness among older adults in the Netherlands after 2007, countries that in a given period did not experience the same policy changes as the treated unit can be used as a “donor pool” to create the control group for both SCM and DiD. The first wave is collected in the 2004 and it represents the pre-treatment period, while the other two waves are collected after the policy intervention and they are used for the post-treatment estimation.

The SHARE data include older individuals and their partners, who live either in their own house or in a nursing home. As household help is available only for people who live in their own home, we exclude individuals who live in a nursing home.

Loneliness is measured by a one item question which is asked in all three waves: “How often did you feel lonely during the last 12 months?”. The answers are on a three level Likert scale and include the following categories: “hardly ever or never”, “sometimes” and “often”. We have constructed a binary indicator variable where categories “often” and “sometimes” are coded 1 (lonely) while category “hardly ever or never” is coded as 0. For the DiD, this indicator will be our outcome variable. We also use this indicator to estimate the prevalence of loneliness for each of the countries in the four waves. This will be our outcome variable for the SCM.

As predictors we use the same set of covariates in both the SCM and the DiD: gender, marital status, being a migrant, household size, age, number of children, type of settlement, being depressed (measured by the standardized EURO-D multi-level scale), number of chronic diseases, level of mobility (measured by The Global Activity Limitation Index) and using help from others. There was no statistical significant difference in age between waves (67.36 ± 13.7 before 2011 to 68.55 ± 9.24 after 2011, *p* = 0.01). For the depression scale we use a binary indicator (depressed – not depressed) provided by SHARE data. The indicator is calculated based on cut-off points for the EURO-D scale. For the number of chronic diseases we have created a binary indicator coded as yes if number of chronic diseases is one or more; otherwise this variable is 0. Regarding the association between the number of chronic diseases and loneliness - we did not find a statistically significant correlation. The level of mobility is also provided as a binary indicator (limited – not limited) within SHARE data. It is calculated by using The Global Activity Limitation Index that is a one question instrument. Recent studies show that older women report higher levels of loneliness than older men, while also older migrants have a higher probability to experience loneliness than older people living in their country of birth [19]. Older people who live with their partner or with other family members less often report loneliness [16]. Also, older adults who use help of others including their social network report loneliness less often. We also wanted to include education as a potential predictor. However, this variable is measured using different classifications in different waves of the SHARE data and we were therefore unable to use this variable. In both analyses, we use the Netherlands as the treated unit while the other 9 countries are used to construct the control group.

### Synthetic control method and differences in differences

DiD has been widely applied in the evaluation of health policy measures and health interventions [20]. This approach uses observational longitudinal data to simulate an experimental design. It calculates the difference in outcome measures for the treatment group before and after the treatment. The same difference is calculated for the control group. After that the difference within two differences is estimated. In our case, DiD will first estimate the difference in loneliness among older individuals in the Netherlands and those from 9 control countries for the period until 2007. After this, the same difference is calculated for the period 2011–2013. The difference between two differences gives us the average treatment on the treated (ATT) effect and its statistical significance. DiD also allows us to estimate the ATT with a binary outcome variable.

The main disadvantage of DiD is that this approach is based on the very strict “parallel trend assumption” which assume that the average outcomes for control and treatment groups on the outcome measure would follow the same parallel trend over time in the absence of the policy intervention [18]. In other words, it assumes the unobserved confounders that affect the outcome measure do not change over time. However, this assumption is not always plausible when it comes to the evaluation of health policy interventions [22].

Recently Abadie et al., 2015 has suggested SCM to evaluate policy interventions. The SCM compares results on the outcome variable between the treated unit (one country or region) with its counterfactual outcome. The counterfactual outcome is calculated by using the weighted average of the outcomes from several control units (synthetic control group) that were not exposed to the policy measure. The policy effect (treatment effect) is calculated as the difference in the outcome variable between the treated unit and the synthetic control group (control group) after the policy implementation [15]. In this way SCM incorporates advantages from DiD (comparing the control and treated groups before and after the intervention) and propensity scores matching (the synthetic control group is constructed as an average of several control units that are matched on a set of covariates in order to be the most similar with the treated unit) [21]. In other words, by SCM we compare the loneliness among older adults in The Netherlands and the synthetic control group for the Netherlands (control group constructed in a way to be most similar to the Netherlands) before and after policy change. The SCM also does not require a “parallel trend assumption”- the effects of unobserved cofounding factors can vary within time. The main disadvantage of this method is that it is applied using only one treated unit [15]. In order to provide more robust results, we will use both approaches.

To obtain the level of statistical significance of the treatment effect, Abadie et al. (2015) suggested the use of a placebo – test. The placebo - test represents a permutation in which each control group is used as if it was exposed to the treatment and the treatment unit is excluded. In this study we choose two countries with similar trends in loneliness as the Netherlands and use them as potential treatment units in the placebo tests.

## Results

In Table 2 we present figures on the prevalence of loneliness in different European countries. The prevalence of loneliness is higher in 2011–2013 than in 2004 in all countries. It is observed that loneliness is lowest in the Denmark, while the highest prevalence is reported in Italy. We present descriptive statistics for all variables included in the study (Appendix 1, Additional file 1). The data are presented for the Netherlands (treated group) and other 9 countries (control group) first for the pre-treatment period (wave 1) and then for the post-treatment period (wave 4 and wave 5).

**Table 2:** The prevalence of loneliness in different European countries

	2004	2011	2013
Austria	7.5%	17.7%	17.1%
Belgium	11.6%	28.8%	25.0%
Denmark	3.7%	11.0%	10.0%
Germany	6.8%	16.8%	16.8%
France	13.9%	26.1%	30.2%
Italy	15.4%	31.4%	33.4%
The Netherlands	6.5%	16.0%	22.0%
Spain	12.3%	20.1%	21.4%
Sweden	6.1%	24.0%	22.1%
Switzerland	4.2%	12.7%	14.1%
Total	9.0%	21.3%	21.7%

**Fig. 1:**
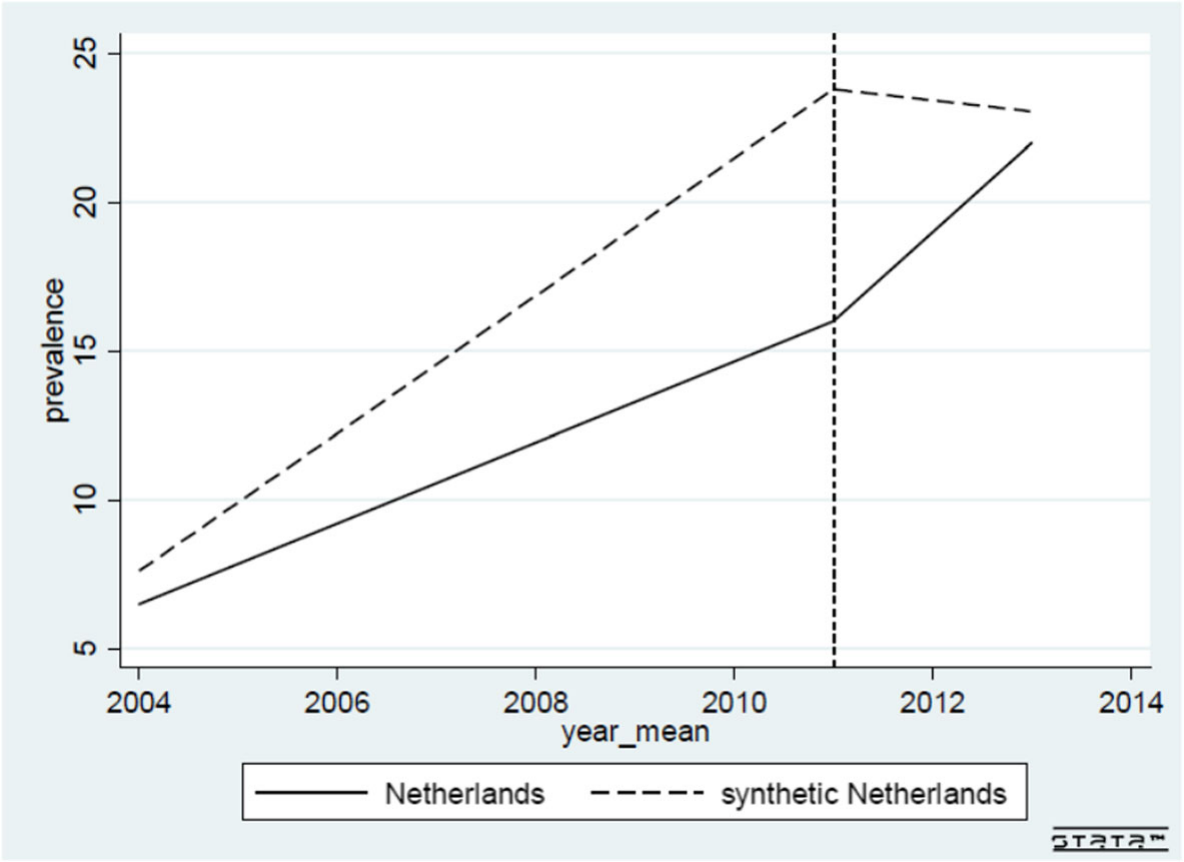
Trends in prevalence of loneliness among older adults in the Netherlands and control group

Next, we present the results of the SCM estimations. Figure 1 presents the trend in the prevalence of loneliness among older adults in the Netherlands (bold line) and in the synthetic control group for the Netherlands (dashed line). The trend in general shows a lower prevalence of loneliness in the Netherlands than in the synthetic control group during the whole period 2004–2013. After 2011 loneliness in the Netherlands increased (from 6.5% in 2004 to 22.0% in 2013). However, in 2013 the prevalence of loneliness in the Netherlands is still lower than in the synthetic control group for the Netherlands (23.5%). From the SCM estimation we cannot say whether this difference is statistically significant. Table 3 presents the combination of predictors for both the treated unit and the synthetic control group. We also present root mean square predicted error (RMSPE) for the real prevalence of loneliness among older adults. This measure shows that the overall fit of the used covariates is good. We present weights for each of the countries included in the study (see Appendix 2, Additional file 1) and we present placebo tests in Appendix 3, Additional file 1. To perform the placebo tests, we use Germany and Sweden as potentially treated units, while the other countries are used as donor pools. The results from the placebo tests differ from the results for the Netherlands (see Appendix 3, Additional file 1).

**Table 3:** Pre-treatment characteristics (averages) for the Netherlands and synthetic Netherlands

	The Netherlands	Synthetic NL
Gender (1-male; 2-female)	1.51	1.52
Marital status (1-married/living together; 0- -not married/separated, divorced/widowed)	0.84	0.83
Being a foreigner (1-yes; 0-no)	0.94	0.92
Received help from others(1-yes; 0-no)	0.24	0.23
Number of children (from 0 up to 17)	2.46	2.19
Type of settlement (1-urban; 0-rural)	0.78	0.73
Age (from 55 up to 104)	66.16	67.32
Household size (from 1up to 10)	1.97	1.98
Depressed (1-yes, 0-no)	0.18	0.21
Mobility (1-limited, 0-no-limitation)	0.45	0.44
Presence of chronic diseases (1-yes, 0-no)	0.71	0.76
RMSPE (root mean square predicted error)	1.11	

Table 4 present the results of the DiD estimations. Here, we compare the reported loneliness among older adults in the Netherlands and in the control group consisting of the 9 other countries before and after the policy change in 2007. Furthermore, to check the robustness of our results, we have applied DiD to compare the Netherlands with each control country separately. Results from the DiD estimator show that there is no statistically significant difference in loneliness between the treated and the control group before and after the treatment when we use all 9 countries in the control group (see Table 4)**.**We also present the results where each control country is compared separately with the Netherlands. The results are similar: there are no statistically significant differences in loneliness between the Netherlands and each of the control countries. Also, we report balancing t-tests for each of the DiD models (see Appendix 4, Additional file 1). The balancing tests show that for all models (when all 9 countries are used as control group and when each of the country is used separately as a control group) there is a significant difference in almost all covariates between the Netherlands and the control group during the pre-treatment period. Since we have only one pre-treatment period, we present the trends in loneliness comparing the Netherlands with each of the control countries only for year 2004. The results from the graphs differ between countries and show that the parallel time trend holds if Germany and Italy are used as control group (see Appendix 5, Additional file 1).

**Table 4:** DiD estimators using different control countries

	9 control countries		Sweden as control country		Denmark as a control country		Germany as a control country		Italy as a control country		Spain as a control country	
	B	SE	B	SE	B	SE	B	SE	B	SE	B	SE
Gender	0.030*	0.07	0.023	0.017	0.009	0.019	0.039*	0.018	0.044*	0.017	0.040**	0.016
Marital status	−0.199*	0.008	−0.263*	0.025	−0.223*	0.016	−0.260*	0.021	−0.238*	0.022	−0.218*	0.020
Being a foreigner	−0.027*	0.012	−0.029	0.033	−0.116**	0.004	0.043	0.028	−0-083**	0.007	−0.082**	0.040
Received help from others	0.030*	0.008	−0.015	0.020	0.012	0.021	0.049*	0.021	0.033	0.021	0.038	0.020
Number of children	−0.006*	0.002	0.002	0.006	−0.001	0.006	−0.008	0.006	−0.007	0.006	0.000	0.005
Type of settlement	0.007	0.007	0.006	0.021	0.004	0.022	0.019	0.20	0.024	0.018	0.013	0.023
Age	0.000	0.000	−0.001	0.010	−0.001	0.001	−0.001	0.001	0.001	0.001	−0.002**	0.001
Household size	−0.037*	0.04	−0.069*	0.016	−0.077*	0.017	−0.062*	0.015	−0.031*	0.011	−0.049*	0.008
Depressed	0.239*	0.008	0.262*	0.021	0.259*	0.024	0.207*	0.022	0.254*	0.011	0.260*	0.018
			9 control countries		Sweden as control country		Denmark as a control country		Germany as a control country		Italy as a control country	
	B	SE	B	SE	B	SE	B	SE	B	SE	B	SE
Presence of chronic diseases	0.009	0.008	0.30	0.20	−0.015	0.023	0.006	0.022	0.025	0.020	0.009	0.020
Mobility	0.043*	0.007	0.032	0.018	0.058*	0.020	0.058	0.019	0.059*	0.018	0.030	0.017
	Difference	SE	Difference	SE	Difference	SE	Difference	SE	Difference	SE	Difference	SE
Before	−0.017	0.014	0.05	0.018	0.05	0.020	−0.02	0.020	−0.09	0.021	−0.03	0.021
After	−0.050	0.025	−0.28	0.116	−0.11	0.110	0.06	0.162	−0.18	0.031	−0.05	0.029
DiD (ATT)	−0.033	0.028	−0.033	0.117	−0.17	0.112	0.08	0.163	−0.09	0.036	−0.02	0.034
R^2^	0.17		0.19		0.20		0.19		0.21		0.21	

*p < 0.01

**p < 0.05

## Discussion

The aim of this paper was to assess to what extent changes in the policy related to household help through the introduction of a new law (the Wmo) had influenced the level of loneliness among older adults. The results from the SCM show that the prevalence of loneliness among older adults in the Netherlands increased during the period 2004–2013. This is observed in Fig. 1 – which shows an increase in loneliness among older adults in the Netherlands after 2011. However, it is difficult to estimate to what extent this change is significant and to what extent it can be attributed to the policy change. The SCM graph shows that the prevalence of loneliness in the Netherlands is increasing but is still lower than in the other 9 European countries. This is similar with the situation before the policy change - in absolute numbers of the prevalence of loneliness in the Netherlands in 2004 is lower than in the control countries [5]. When we use DiD and use the same countries as in the SCM, results are similar. They show that there is no significant difference in the incidence of loneliness between older adults in the Netherlands and those in other control countries. This implies that the incidence of loneliness in the Netherlands did not change significantly after the policy change. To check the robustness of the results, we have also performed DiD analyses using each of 9 countries (Austria, Belgium, Germany, Sweden, Italy, Spain, Switzerland, France and Denmark) as separate control groups The results of the test on the parallel trend show that Germany and Italy are the most suitable control countries. When we use Germany and Italy as controls, the DiD estimator is not statistically significant. This can be seen as a lack of treatment effect – in other words the policy change did not provoke a change in loneliness. On the other side, the results of the SCM show that there is an increasing trend in loneliness among Dutch elderly after 2011, but that this level is still lower than in synthetic control group for the Netherlands (in control countries). This may imply that the effects of the changes in the household help policy will only be visible over a longer period of time.

Our results also show that both approaches do have some advantages and disadvantages. The DiD estimator has more statistical power since it is based on a larger number of observations. However, the DiD estimator focuses on two time points only – before and after policy changes occur. The SCM is based on aggregated data and lacks a statistic parameter (such as ATT in DiD). On the other side, the SCM shows the trend in the changes related to the outcome variable during the whole observed period. For the evaluation of community based interventions that are expected to have effects after a longer period of time and where the parallel assumption of the DiD estimator might be questionable, both approaches could be useful.

## Conclusion

Our results do not suggest that the transfer of household help to the municipalities through the introduction of the Wmo has had an effect on loneliness. Based on our evidence, the increase in the prevalence of loneliness since 2011 cannot be attributed to the policy change. One of the aims of the new law – the Wmo – was to ensure the active social participation of everyone in society. The increase in loneliness in recent years can be seen as a failure of this policy objective. Older adults who feel lonely are less likely to actively participate in society. The Wmo emphasizes self-reliance, which means that older adults should use their own resources in order to prevent disruption in quality of life such as loneliness.

Within the Wmo the responsibility for household help is given to local municipalities. They are expected to be able to recognize better and deal with the needs of their citizens. One goal of the Wmo was better targeting of eligible persons and improved efficiency in service delivery. Some recent studies show that users are satisfied with the care provided within the Wmo [13]. The introduction of the Wmo has also been accompanied by large budget cuts. This suggests that the introduction of the Wmo has not been a complete failure. Furthermore, we have evaluated here the household help – an intervention which main goal is to provide help in the household. Preventing loneliness by using household help is a worthy objective but also just a secondary goal of this law.

The prevalence of loneliness appears to have increased in all other 9 European countries, which might be related to the fact that older adults are nowadays more willing to acknowledge they are lonely than in the past.

This study also has some limitations. We use a cross-country comparison to examine the effects of changes in household help. However, there are big differences between municipalities in the organization and type of services that are provided. In this study we were not able to compare the different effects on policy changes between different municipalities and regions in the Netherlands. Also, this study is limited by the fact that we use only two years (2011 and 2013) to estimate the post-treatment effects. Future research should estimate the effects of changes in household help using data over a longer period of time. As a pre-treatment period, we also use only one wave. This gives us limited possibilities to test the parallel trend assumption within the DiD approach.

The measurement of loneliness is also a potential limitation. Previous studies suggest that a one item scale: “Do you feel lonely?” with multi-level answers has high reliability [7]. However, for a better effect evaluation study, it would be useful to compare the prevalence data obtained using a one item scale with data obtained from multi-item scale such as longer version of R-UCLA Loneliness Scale.

## Additional file

Additional file 1: Appendix 1: Descriptive statistics for all variables included in the study, Appendix 2: Countries and their loadings to synthetic the Netherlands, Appendix 3: placebo tests related to SCM, Appendix 4: Balancing t-tests for each of the DiD models, Appendix 5: Pre- treatment tests for DiD estimator. (DOCX 157 kb)

## Abbreviations

ATT: Average treatment on treated
AWBZ: General Law on Exceptional Medical Expenses
DiD: Difference-in-differences
RMSPE: Mean square predicted error
SCM: Synthetic control method
SHARE: The Survey of Health, Ageing and Retirement in Europe
Wmo: Social Support Act
