# Supplementary material for: Does household help prevent loneliness among the elderly? An evaluation of a policy reform in the Netherlands
Source: BMC Public Health. 2018 Sep 10;18:1104. doi: 10.1186/s12889-018-6004-6 (PMC6131846; doi:10.1186/s12889-018-6004-6)
Supplement: Supplementary file 1 — Appendix 1: Descriptive statistics for all variables included in the study, Appendix 2: Countries and their loadings to synthetic the Netherlands, Appendix 3: placebo tests related to SCM, Appendix 4: Balancing t-tests for each of the DiD models, Appendix 5: Pre- treatment tests for DiD estimator. (DOCX 157 kb) [file 12889_2018_6004_MOESM1_ESM.docx]

| Appendix 1: Descriptive statistics for all variables included in the study | | | | | | |
| --- | --- | --- | --- | --- | --- | --- |
|  | The Netherlands | | Other countries | | Total sample | |
|  | Pre-treatment  Mean (SD) | After treatment  Mean (SD) | Pre-treatment  Mean (SD) | After treatment  Mean (SD) | Pre-treatment  Mean (SD) | After treatment  Mean (SD) |
| Loneliness (binary indicator used as outcome in DiD) | 0.28 (0.45) | 0.28(0.39) | 0.33 (0.47) | 0.22 (0.41) | 0.33 (0.46) | 0.22 (0.41) |
| Gender (2-female; 1-male) | 1.5 (0.50) | 1.54 (0.49) | 1.54 (0.49) | 1.54 (0.49) | 1.54 (0.49) | 1.54 (0.49) |
| Country of origin (1=born in a country of interview; 0=born outside country of interview) | 0.95 (0.22) | 0.93 (0.25) | 0.91 (0.28) | 0.91 (0.28) | 0.92 (0.27) | 0.91 (0.28) |
| Age (from 55 to 104) | 66.16 (8.64) | 67.73 (8.59) | 67.52 (8.92) | 68.63 (9.18) | 67.36 (8.89) | 68.55 (9.14) |
| Number of children (from 0 to 17) | 2.46 (1.69) | 2.34 (1.43) | 2.16 (1.53) | 2.14 (1.44) | 2.19 (1.55) | 2.16 (1.44) |
| Household size (1 to 10) | 1.98 (0.67) | 1.93 (0.65) | 2.04 (0.87) | 2.03 (0.85) | 2.04 (0.85) | 2.02 (0.84) |
| Type of settlement (1-urban; 0-rural) | 0.79 (0.41) | 0.78 (0.41) | 0.75 (0.43) | 0.66 (0.47) | 0.75 (0.42) | 0.68 (0.47) |
| Marital status (1-married; 0 non-married | 0.85 (0.35) | 0.79 (0.41) | 0.77 (0.42) | 0.73 (0.44) | 0.78 (0.41) | 0.73 (0.44) |
| Help received from others (1-yes; 0-no) | 0.25 (0.43) | 0.18 (0.38) | 0.24 (0.42) | 0.19 (0.39) | 0.24 (0.43) | 0.19 (0.39) |
| Depressed (1-yes; 0-no) | 0.19 (0.39) | 0.17 (0.37) | 0.25 (0.43) | 0.26 (0.44) | 0.25 (0.43) | 0.25 (0.43) |
| Presence of chronic diseases (1-yes; 0-no) | 0.71 (0.45) | 0.73 (0.44) | 0.78 (0.41) | 0.78 (0.41) | 0.78 (0.41) | 0.78 (0.41) |
| Mobility (1-limited; 0-non-limited) | 0.45 (0.49) | 0.51 (0.50) | 0.46 (0.49) | 0.44 (0.49) | 0.46 (0.48) | 0.45 (0.49) |

*^* Pre-treatment – includes data from wave 1 collected in 2004^*

*_** After treatment – includes data from wave 4 and wave 5 collected in 2011 and 2013._*

**Appendix 2:**

| Table 1: Countries and their loadings to synthetic the Netherlands | |
| --- | --- |
| Country | Unit weight |
| Austria | 0.000 |
| Belgium | 0.000 |
| Germany | 0.043 |
| Denmark | 0.000 |
| Italy | 0.182 |
| France | 0.000 |
| Sweden | 0.665 |
| Spain | 0.000 |
| Switzerland | 0.110 |

**Appendix 3 – placebo tests related to SCM**

Figure 2: Placebo test with Sweden as treated unit

Figure 3: Placebo test when Germany is used as a treated unit

| T-test for period = 0 country Austria (control) and the Netherlands (treated) | | | | |
| --- | --- | --- | --- | --- |
|  | Mean control | Mean treated | Difference | t-test |
| Loneliness | 0.221 | 0.241 | 0.020 | 2.36* |
| Gender (2-female; 1-male) | 1.573 | 1.531 | -0.042 | 4.44* |
| Country of origin (1=born in a country of interview; 0=born outside country of interview) | 0.914 | 0.940 | 0.026 | 3.37* |
| Age (from 55 to 104) | 68.309 | 67.219 | -1.091* | 6.57* |
| Number of children (from 0 to 17) | 2.017 | 2.381 | 0.363 | 10.69* |
| Household size (1 to 10) | 1.913 | 1.948 | 0.036 | 2.40* |
| Type of settlement (1-urban; 0-rural) | 0.640 | 0.780 | 0.140 | 16.20* |
| Marital status (1-married; 0 non-married) | 0.659 | 0.831 | 0.172 | 14.16* |
| Help received from others (1-yes; 0-no) | 0.216 | 0.214 | -0.002 | 0.16 |
| Depressed (1-yes; 0-no) | 0.196 | 0.176 | -0.020 | 2.66* |
| Presence of chronic diseases (1-yes; 0-no) | 0.765 | 0.728 | -0.037 | 4.47* |
| Mobility (1-limited; 0-non-limited) | 0.482 | 0.481 | -0.001 | 0.15 |
| **p<0.05* | | | | |

**Appendix 4 - balancing t-tests for each of the DiD models**

| T-test for period = 0 country Belgium (control) and the Netherlands (treated) | | | | |
| --- | --- | --- | --- | --- |
|  | Mean control | Mean treated | Difference | t-test |
| Loneliness | 0.290 | 0.241 | -0.049 | 5.95* |
| Gender (2-female; 1-male) | 1.540 | 1.531 | -0.009 | 1.04 |
| Country of origin (1=born in a country of interview; 0=born outside country of interview) | 0.909 | 0.940 | 0.031 | 5.03* |
| Age (from 55 to 104) | 67.807 | 67.819 | -0.588 | 3.77* |
| Number of children (from 0 to 17) | 2.132 | 2.381 | 0.249 | 7.85* |
| Household size (1 to 10) | 1.957 | 1.948 | -0.008 | 0.65 |
| Type of settlement (1-urban; 0-rural) | 0.755 | 0.780 | 0.025 | 3.31* |
| Marital status (1-married; 0 non-married) | 0.766 | 0.831 | 0.065 | 7.08* |
| Help received from others (1-yes; 0-no) | 0.244 | 0.214 | -0.030 | 2.98* |
| Depressed (1-yes; 0-no) | 0.266 | 0.176 | -0.090 | 12.26* |
| Presence of chronic diseases (1-yes; 0-no) | 0.807 | 0.728 | -0.079 | 10.75* |
| Mobility (1-limited; 0-non-limited) | 0.449 | 0.481 | 0.031 | 3.57* |
| **p<0.05* | | | | |

| T-test for period =0 country Denmark (control) and the Netherlands (treated) | | | | |
| --- | --- | --- | --- | --- |
|  | Mean control | Mean treated | Difference | t-test |
| Loneliness | 0.124 | 0.241 | 0.116 | 14.60* |
| Gender (2-female; 1-male) | 1.529 | 1.531 |  |  |
| Country of origin (1=born in a country of interview; 0=born outside country of interview) | 0.968 | 0.940 | -0.028 | 5.14* |
| Age (from 55 to 104) | 67.425 | 67.219 | -0.207 | 1.19 |
| Number of children (from 0 to 17) | 2.215 | 2.381 | 0.166 | 4.92* |
| Household size (1 to 10) | 1.832 | 1.948 | 0.116 | 9.17* |
| Type of settlement (1-urban; 0-rural) | 0.779 | 0.780 | 0.001 | 0.05 |
| Marital status (1-married; 0 non-married) | 0.759 | 0.831 | 0.072 | 7.15* |
| Help received from others (1-yes; 0-no) | 0.299 | 0.214 | -0.086 | 6.86* |
| Depressed (1-yes; 0-no) | 0.167 | 0.176 | 0.009 | 1.23 |
| Presence of chronic diseases (1-yes; 0-no) | 0.780 | 0.728 | -0.052 | 6.15* |
| Mobility (1-limited; 0-non-limited) | 0.399 | 0.481 | 0.082 | 8.38* |
| **p<0.05* | | | | |

| T-test for period =0 country France (control) and the Netherlands (treated) | | | | |
| --- | --- | --- | --- | --- |
|  | Mean control | Mean treated | Difference | t-test |
| Loneliness | 0.324 | 0.241 | -0.083 | 9.29* |
| Gender (2-female; 1-male) | 1.565 | 1.531 | -0.034 | 3.77* |
| Country of origin (1=born in a country of interview; 0=born outside country of interview) | 0.869 | 0.940 | 0.071 | 9.63* |
| Age (from 55 to 104) | 68.606 | 68.219 | -1.387 | 8.34* |
| Number of children (from 0 to 17) | 2.227 | 2.381 | 0.154 | 4.53* |
| Household size (1 to 10) | 1.906 | 1.948 | 0.043 | 3.20* |
| Type of settlement (1-urban; 0-rural) | 0.587 | 0.780 | 0.193 | 23.03* |
| Marital status (1-married; 0 non-married) | 0.722 | 0.831 | 0.109 | 10.51* |
| Help received from others (1-yes; 0-no) | 0.194 | 0.214 | 0.021 | 2.13* |
| Depressed (1-yes; 0-no) | 0.339 | 0.176 | -0.164 | 20.65* |
| Presence of chronic diseases (1-yes; 0-no) | 0.798 | 0.728 | -0.070 | 9.06* |
| Mobility (1-limited; 0-non-limited) | 0.441 | 0.483 | 0.038 | 4.16* |
| **p<0.05* | | | | |

| T-test for period =0 country Germany (control) and the Netherlands (treated) | | | | |
| --- | --- | --- | --- | --- |
|  | Mean control | Mean treated | Difference | t-test |
| Loneliness | 0.207 | 0.241 | 0.034 | 4.27* |
| Gender (2-female; 1-male) | 1.516 | 1.531 | 0.014 | 1.60 |
| Country of origin (1=born in a country of interview; 0=born outside country of interview) | 0.836 | 0.940 | 0.104 | 15.10* |
| Age (from 55 to 104) | 67.517 | 67.219 | -0.0299 | 1.96 |
| Number of children (from 0 to 17) | 1.951 | 2.381 | 0.429 | 14.03* |
| Household size (1 to 10) | 1.989 | 1.948 | -0.040 | 3.35* |
| Type of settlement (1-urban; 0-rural) | 0.615 | 0.780 | 0.165 | 2.23* |
| Marital status (1-married; 0 non-married) | 0.800 | 0.831 | 0.031 | 3.75* |
| Help received from others (1-yes; 0-no) | 0.271 | 0.214 | -0.057 | 4.80* |
| Depressed (1-yes; 0-no) | 0.217 | 0.176 | -0.041 | 5.79* |
| Presence of chronic diseases (1-yes; 0-no) | 0.820 | 0.728 | -0.092 | 12.48* |
| Mobility (1-limited; 0-non-limited) | 0.523 | 0.481 | -0.042 | 4.75* |
| **p<0.05* | | | | |

| T-test for period =0 country Italy (control) and the Netherlands (treated) | | | | |
| --- | --- | --- | --- | --- |
|  | Mean control | Mean treated | Difference | t-test |
| Loneliness | 0.351 | 0.241 | -0.110 | 12.55 |
| Gender (2-female; 1-male) | 1.537 | 1.531 | -0.006 | 0.67 |
| Country of origin (1=born in a country of interview; 0=born outside country of interview) | 0.988 | 0.940 | -0.048 | 10.99* |
| Age (from 55 to 104) | 68.177 | 67.219 | -0.958 | 6.07* |
| Number of children (from 0 to 17) | 1.986 | 2.381 | 0.395 | 12.30* |
| Household size (1 to 10) | 2.400 | 1.948 | -0.451 | 28.41* |
| Type of settlement (1-urban; 0-rural) | 0.599 | 0.780 | 0.181 | 21.57* |
| Marital status (1-married; 0 non-married) | 0.780 | 0.831 | 0.051 | 5.46* |
| Help received from others (1-yes; 0-no) | 0.154 | 0.214 | 0.061 | 6.12* |
| Depressed (1-yes; 0-no) | 0.352 | 0.176 | -0.176 | 22.18* |
| Presence of chronic diseases (1-yes; 0-no) | 0.787 | 0.728 | -0.059 | 7.61* |
| Mobility (1-limited; 0-non-limited) | 0.444 | 0.481 | 0.037 | 4.05* |
| **p<0.05* | | | | |

| T-test for period =0 country Sweden (control) and the Netherlands (treated) | | | | |
| --- | --- | --- | --- | --- |
|  | Mean control | Mean treated | Difference | t-test |
| Loneliness | 0.231 | 0.241 | 0.019 | 1.24 |
| Gender (2-female; 1-male) | 1.527 | 1.531 | 0.003 | 0.37 |
| Country of origin (1=born in a country of interview; 0=born outside country of interview) | 0.919 | 0.940 | 0.021 | 3.74* |
| Age (from 55 to 104) | 68.692 | 67.219 | -1.473 | 9.40* |
| Number of children (from 0 to 17) | 2.279 | 2.381 | 0.101 | 3.28* |
| Household size (1 to 10) | 1.851 | 1.948 | 0.098 | 8.91* |
| Type of settlement (1-urban; 0-rural) | 0.825 | 0.780 | -0.046 | 6.30* |
| Marital status (1-married; 0 non-married) | 0.808 | 0.831 | 0.023 | 2.67* |
| Help received from others (1-yes; 0-no) | 0.223 | 0.214 | -0.009 | 0.84 |
| Depressed (1-yes; 0-no) | 0.183 | 0.176 | -0.007 | 1.04 |
| Presence of chronic diseases (1-yes; 0-no) | 0.782 | 0.728 | -0.054 | 7.00* |
| Mobility (1-limited; 0-non-limited) | 0.424 | 0.481 | 0.056 | 6.33* |
| **p<0.05* | | | | |
| T-test for period =0 country Spain (control) and the Netherlands (treated) | | | | |
|  | Mean control | Mean treated | Difference | t-test |
| Loneliness | 0.243 | 0.241 | -0.003 | 0.34 |
| Gender (2-female; 1-male) | 1.538 | 1.531 | -0.007 | 0.86 |
| Country of origin (1=born in a country of interview; 0=born outside country of interview) | 0.957 | 0.940 | -0.017 | 3.45* |
| Age (from 55 to 104) | 70.031 | 60.219 | -2.813 | 17.36* |
| Number of children (from 0 to 17) | 2.352 | 2.381 | 0.029 | 0.85 |
| Household size (1 to 10) | 2.379 | 1.948 | -0.430 | 27.80* |
| Type of settlement (1-urban; 0-rural) | 0.934 | 0.780 | -0.155 | 26.54* |
| Marital status (1-married; 0 non-married) | 0.790 | 0.831 | 0.041 | 4.74* |
| Help received from others (1-yes; 0-no) | 0.157 | 0.214 | 0.057 | 5.72* |
| Depressed (1-yes; 0-no) | 0.324 | 0.176 | -0.148 | 19.48* |
| Presence of chronic diseases (1-yes; 0-no) | 0.835 | 0.728 | -0.107 | 15.31* |
| Mobility (1-limited; 0-non-limited) | 0.429 | 0.481 | 0.052 | 5.97* |
| **p<0.05* | | | | |

| T-test for period =0 country Switzerland (control) and the Netherlands (treated) | | | | |
| --- | --- | --- | --- | --- |
|  | Mean control | Mean treated | Difference | t-test |
| Loneliness | 0.164 | 0.241 | 0.077 | 8.46* |
| Gender (2-female; 1-male) |  |  |  |  |
| Country of origin (1=born in a country of interview; 0=born outside country of interview) | 0.828 | 0.940 | 0.112 | 10.50* |
| Age (from 55 to 104) | 1.534 | 1.531 | -0.003 | 0.30 |
| Number of children (from 0 to 17) | 2.040 | 2.381 | 0.341 | 9.05* |
| Household size (1 to 10) | 1.961 | 1.948 | -0.012 | 0.83 |
| Type of settlement (1-urban; 0-rural) | 0.447 | 0.780 | 0.333 | 34.12* |
| Marital status (1-married; 0 non-married) | 0.750 | 0.831 | 0.081 | 5.82* |
| Help received from others (1-yes; 0-no) | 0.152 | 0.214 | 0.063 | 5.94* |
| Depressed (1-yes; 0-no) | 0.172 | 0.176 | 0.004 | 0.43 |
| Presence of chronic diseases (1-yes; 0-no) | 0.661 | 0.728 | 0.067 | 6.82* |
| Mobility (1-limited; 0-non-limited) | 0.313 | 0.481 | 0.167 | 16.00* |
| **p<0.05* | | | | |
| T-test for period =0 all 9 countries (controls) and the Netherlands (treated) | | | | |
|  | Mean control | Mean treated | Difference | t-test |
| Loneliness | 0.246 | 0.241 | -0.005 | 0.78 |
| Gender (2-female; 1-male) | 1.539 | 1.531 | 0.009 | 1.27 |
| Country of origin (1=born in a country of interview; 0=born outside country of interview) | 0.913 | 0.940 | 0.027 | 5.47* |
| Age (from 55 to 104) | 68.367 | 67.219 | -1.148 | 9.19* |
| Number of children (from 0 to 17) | 2.142 | 2.381 | 0.238 | 9.77* |
| Household size (1 to 10) | 2.039 | 1.948 | -0.090 | 7.84* |
| Type of settlement (1-urban; 0-rural) | 0.705 | 0.780 | 0.074 | 11.88* |
| Marital status (1-married; 0 non-married) | 0.774 | 0.831 | 0.057 | 7.93* |
| Help received from others (1-yes; 0-no) | 0.209 | 0.214 | 0.005 | 0.62 |
| Depressed (1-yes; 0-no) | 0.255 | 0.176 | -0.079 | 13.21* |
| Presence of chronic diseases (1-yes; 0-no) | 0.791 | 0.728 | -0.063 | 11.09* |
| Mobility (1-limited; 0-non-limited) | 0.441 | 0.481 | 0.040 | 5.70* |
| **p<0.05* | | | | |

**Appendix 5 – pre- treatment tests for DiD estimator**

Graph 1: Pre-treatment trends in loneliness using The Netherlands as treated and Sweden as control group

Graph 2: Pre-treatment trends in loneliness using The Netherlands as treated and Germany as control group

Graph 3: Pre-treatment trends in loneliness using The Netherlands as treated and Italy as control group

Graph 4: Pre-treatment trends in loneliness using The Netherlands as treated and Spain as control group

Graph 5: Pre-treatment trends in loneliness using The Netherlands as treated and Denmark as control group
